# Serum Adropin as a Potential Biomarker for Predicting the Development of Type 2 Diabetes Mellitus in Individuals With Metabolic Dysfunction-Associated Fatty Liver Disease

**DOI:** 10.3389/fphys.2021.696163

**Published:** 2021-07-22

**Authors:** Na Li, Guomin Xie, Biao Zhou, Aijuan Qu, Hua Meng, Jia Liu, Guang Wang

**Affiliations:** ^1^Department of Endocrinology, Beijing Chao-Yang Hospital, Capital Medical University, Beijing, China; ^2^Department of Physiology and Pathophysiology, School of Basic Medical Sciences, Capital Medical University, Beijing, China; ^3^Key Laboratory of Remodeling-Related Cardiovascular Diseases, Ministry of Education, Beijing, China; ^4^General Surgery Department and Obesity and Metabolic Disease Center, China-Japan Friendship Hospital, Beijing, China

**Keywords:** metabolic dysfunction-associated fatty liver disease, adropin, triglyceride, steatohepatitis, type 2 diabetes mellitus

## Abstract

**Background:** Adropin, a peptide translated from the *Energy Homeostasis Associated gene* (*ENHO*), was mainly expressed in the liver and was a regulator in metabolic and energy homeostasis. This study aims to investigate the correlation between adropin and histological characteristics of the liver, and the clinical relevance of adropin in patients with metabolic dysfunction-associated fatty liver disease (MAFLD).

**Methods:** A total of 62 subjects, including 32 healthy controls and 30 MAFLD patients, were enrolled in this case-control study. The MAFLD patients were further divided into two subgroups, including NGT-M group and T2DM-M group. Serum adropin levels, metabolic parameters and intrahepatic lipids, the liver *ENHO* mRNA expressions and histological characteristics were investigated.

**Results:** MAFLD patients showed significantly lower circulating adropin compared with healthy controls (2.02 ± 2.92 vs. 5.52 ± 0.65 ng/mL, *P* < 0.0001). Subgroup analysis exhibited dramatically declined serum adropin levels in T2DM-M patients compared with NGT-M group (0.51 ± 0.73 vs. 4.00 ± 3.52 ng/mL, *P* < 0.001). H&E and Oil Red O staining show exacerbated steatohepatitis in T2DM-M patients in contrast with NGT-M group. Furthermore, serum adropin concentrations were negatively correlated with intrahepatic triglyceride (TG), total cholesterol (TC), and NAFLD activity score (NAS) (TG: *r* = −0.495; TC: *r* = −0.392; NAS: *r* = −0.451; all *P* < 0.05).

**Conclusions:** MAFLD patients showed significantly lower adropin levels than the healthy controls, especially in T2DM patients. Adropin maybe a potential biomarker for predicting the development of MAFLD, especially in T2DM individuals.

## Introduction

Metabolic dysfunction-associated fatty liver disease (MAFLD), formerly named non-alcoholic fatty liver disease (NAFLD), affecting a quarter of the general population, which has become the most common liver disease (Sarin et al., 2020; Eslam et al., 2020b). The newly proposed diagnostic criteria for MAFLD irrespective of alcohol intake or other concomitant liver diseases. The criteria are principally based on an evidence of hepatic steatosis and include one of the following three criteria: overweight/obesity, presence of type 2 diabetes mellitus (T2DM), or evidence of metabolic dysfunction (Eslam et al., 2020a). The fundamental pathophysiological change of hepatic steatosis is accumulated free fatty acids (FFAs) and triglycerides (TGs) in hepatocytes. Steatohepatitis is the severely advanced stage of hepatic steatosis and is typically characterized by steatosis, lobular inflammation, and ballooning with or without peri-sinusoidal fibrosis (Castera et al., 2019). Many biomarkers which can be used to predict steatohepatitis in patients with MAFLD have been reported. However, considering the invasiveness and expensive cost of liver biopsy, a noninvasive, economical, easily accessible, highly sensitive, and specific biomarker is urgently needed to predict the development of MAFLD.

Adropin, a secreted peptide encoded by the *Energy Homeostasis Associated* (*ENHO*) gene (Kumar et al., 2008), was mainly expressed in the brain and liver. Recent studies showed beneficial effects of adropin on improving glucose homeostasis, dyslipidemia, obesity-associated hyperinsulinemia, and energy homeostasis. Clinical studies suggested that serum adropin was reduced in many diseases, such as NAFLD, T2DM, diabetic nephropathies, coronary atherosclerosis, hypertension, and polycystic ovary disease (Jasaszwili et al., 2020; Ye et al., 2021). Nevertheless, the correlation between adropin and different glucose tolerance in MAFLD patients remains unclear. On the basis of previous findings, we hypothesized that adropin might be involved in the pathogenesis of MAFLD and the serum adropin could be served as a predictive factor of MAFLD. Thus, this study was designed to investigate the correlation between adropin and histological characteristics of the liver, and the clinical relevance of adropin in MAFLD patients.

## Materials and Methods

### Study Design

From July 2019 to January 2020, 30 MAFLD patients were histologically confirmed with hepatic steatosis, which were enrolled in China-Japan Friendship Hospital. MAFLD patients were divided into two subgroups, including NGT-M group (13 normal glucose tolerance, NGT) and T2DM-M group (17 type 2 diabetes mellitus patients, T2DM). No drug intervention was given in patients with T2DM. We enrolled 32 sex and age matched healthy controls who were free from diagnosed hepatic steatosis based on the results of upper abdomen ultrasonography and routine physical examination at Beijing Chao-yang Hospital Affiliated to Capital Medical University from June 2018 to October 2019. All subjects had to meet the following exclusion criteria: alcohol consumption (≥140 g/wk in men and ≥70 g/wk in women), chronic viral hepatitis, autoimmune hepatitis, drug-induced liver disease, primary biliary cirrhosis, biliary obstruction, Wilson's disease, and α-1 antitrypsin deficiency-associated liver disease. All participants completed a uniform questionnaire containing questions about the histories of present and past illnesses and medical therapy. Thirty MAFLD patients were asked to undergo intra-operative liver biopsy at the time of admission. The study was approved by the human research ethics committee of the China-Japan Friendship Hospital (2019-103-K71-1), according to the principles of the Declaration of Helsinki. Written informed consent was obtained from all subjects.

### Human Liver Tissues and Serum

MAFLD patients (*n* = 30) from the department of general surgery collaboration with China-Japan Friendship Hospital underwent intra-operative liver biopsies during sleeve gastrectomy intervention, following the recommendations by the American Association for the Study of Liver Diseases (Rockey et al., 2009). The histological characteristics of the liver were graded according to a histological scoring system for NAFLD (Kleiner et al., 2005). NAFLD activity score (NAS) was calculated by adding these three items. Serum samples were centrifuged at 3,000 rpm for 15 min, and the obtained serum and liver tissues were immediately snap-frozen and stored at −80°C until use.

### Anthropometric and Biochemical Measurements

Height, weight and waist/hip circumference were measured light clothes on and without shoes, and the body mass index (BMI, kg/m^2^) was calculated. Fasting blood samples were obtained after overnight fast for the measurement of plasma glucose, C-peptide, insulin, alanine aminotransferase (ALT), aspartate aminotransferase (AST), gamma-glutamyl transferase (GGT), total cholesterol (TC), triglyceride (TG), high-density lipoprotein-cholesterol (HDL-c), low-density lipoprotein cholesterol (LDL-c) levels, apoprotein A1 (Apo-A1), apoprotein B (Apo-B), non-esterified fatty acid (NEFA), small dense-low-density lipoprotein cholesterol (sd-LDL). Homeostasis model assessment insulin resistance index (HOMA-IR) was calculated according to the following formula: FINS (μIU/mL) × FBG (mmol/L)/22.5. A 75-g oral glucose tolerance test (OGTT) was performed, plasma glucose levels were measured at 30, 60, 120, and 180 min after oral glucose, the levels of C-peptide and insulin were also detected at 60, 120, and 180 min after oral glucose. Human serum adropin was measured with an enzyme-linked immunosorbent assay (ELISA) kit (Phoenix Pharmaceuticals, Belmont, CA, USA, EK-032-35), according to the manufacturer's protocol.

### Histological Staining

Fresh liver tissue was immediately fixed in 10% phosphate-buffered formalin and 4% neutral-buffered formalin solution for 24 h, embedded in paraffin, sectioned at 6 μm, and then deparaffinized in xylene and rehydrated through a series of decreasing concentrations of ethanol. The sections were prepared for hematoxylin and eosin (H&E) staining, which demarcates fat degeneration. Oil Red O staining was conducted on frozen-sections embedded in OCT to determine hepatic steatosis. Images were captured with a light microscope (Olympus, Tokyo, Japan).

### Liver Lipid Analysis

Hepatic triglycerides and cholesterol, free cholesterol was extracted in 50 mM Tris buffer, homogenized, and incubated at 37°C with shaking overnight. The triglyceride and cholesterol, free cholesterol measurement kit (Solarbio, Beijing, China) were used following the manufacturer's instruction to measure lipid contents.

### RNA Extraction and qRT-PCR

Total RNA was extracted from human liver tissues using Trizol Reagent (Life Technologies, Carlsbad, CA), then reverse transcripted into cDNA with GoScript reverse transcriptase (Promega, Madison, WI), and prepared for RT-qPCR analysis with SYBR green premix (TaKaRa, Nojihigashi, Kusatsu, Shiga, Japan). RT-qPCR assays were performed on CFX Connect Real-Time System (BIO-RAD, CA). Values are expressed as fold change over control, β*-Actin* (*Actb*) was used for normalization to quantify relative mRNA expression levels, relative changes in mRNA expression were calculated using the comparative cycle method (2^−ΔΔCt^). Real-time PCR primer sequences are shown in Supplementary Table 1.

### Statistical Analysis

Normally distributed variables were expressed as mean values ± standard deviation of mean (SD). Non-normally distributed variables were described as medians (25th, 75th percentiles), including TG, FINS and HOMA-IR, NEFA. Categorical variables are expressed as *n* (number) with percentage (%). Comparisons between two groups were performed by Student's *t-*test for continuous variables and χ^2^ analyses for categorical variables. The correlations between adropin and other parameters were analyzed by Pearson's or Spearman's coefficient. Data were analyzed using the IBM SPSS statistical software (version 24.0; SPSS Inc., Chicago, IL, USA) and GraphPad Prism software (version 7.0; CA, USA). Statistically significant was considered at *p* < 0.05 (two-tailed).

## Results

### Clinical Characteristics of the Study Participants

Clinical characteristics including anthropometric parameters and biochemical indexes in both control subjects and MAFLD patients are shown in Table 1. The MAFLD patients had significantly higher levels of BMI, WHR, HbA_1_c, FBG, FINS, HOMA-IR, TG, ALT, AST, and GGT but a lower level of HDL-c compared with healthy controls (All *p* < 0.01). The serum adropin level of MAFLD patients was significantly lower than that of control subjects (2.02 ± 2.92 ng/ml vs. 5.52 ± 0.65 ng/ml, *p* < 0.001).

**Table 1:** Anthropometric parameters and biochemical indexes in healthy controls and MAFLD patients.

	**Control (*n* = 32)**	**MAFLD (*n* = 30)**	***P-*value**
Sex (male), *n* (%)	11 (34)	10 (33)	0.931
Age (years)	35.1 ± 4.8	35.1 ± 9.5	0.989
BMI (Kg/m^2^)	21.46 ± 1.61	36.88 ± 5.79	**<0.001**
Waist circumference (cm)	75.47 ± 8.41	115.30 ± 12.67	**<0.001**
WHR	0.91 ± 0.02	0.95 ± 0.07	**<0.01**
HbA_1_c (%)	5.10 ± 0.44	6.54 ± 1.93	**<0.001**
FBG (mM)	4.74 ± 0.49	7.11 ± 2.84	**<0.001**
FINS (μU/mL)	5.50 (4.90–8.00)	15.89 (12.94–20.32)	**<0.001**
HOMA-IR	1.27 (0.87–1.85)	4.28 (3.76–5.57)	**<0.001**
TC (mM)	4.68 ± 0.59	4.67 ± 0.73	0.950
TG (mM)	0.90 (0.68–1.18)	1.88 (0.94–2.41)	**<0.001**
LDL-C (mM)	2.61 ± 0.56	2.78 ± 0.55	0.307
HDL-C (mM)	1.56 ± 0.33	0.91 ± 0.17	**<0.001**
Apo-A1 (g/L)	1.41 ± 0.23	1.37 ± 0.23	0.592
Apo-B (g/L)	0.78 ± 0.13	0.79 ± 0.15	0.916
sd-LDL (mM)	0.83 ± 0.25	0.79 ± 0.45	0.638
NEFA (mM)	0.49 (0.30–0.57)	0.50 (0.40–0.55)	0.219
ALT (IU/L)	16.75 ± 6.91	51.00 ± 30.52	**<0.001**
AST (IU/L)	19.56 ± 5.00	49.79 ± 25.79	**<0.001**
GGT (IU/L)	15.56 ± 6.20	44.88 ± 37.15	**<0.001**
Adropin (ng/ml)	5.52 ± 0.65	2.02 ± 2.92	**<0.001**

*Values are presented as mean ± SD (standard deviation of mean) for normally distributed data or medians (25th, 75th percentiles) for non-normally distributed data*.

*Apo-A1, apoprotein A1; Apo-B, apoprotein B; BMI, body mass index; FBG, fasting blood glucose; FINS, fasting insulin; HDL-C, high-density lipoprotein cholesterol; HOMA-IR, homeostasis model assessment of insulin resistance; LDL-C, low-density lipoprotein cholesterol; NEFA, non-esterified fatty acid; sd-LDL, small dense-low-density lipoprotein cholesterol; TG, triglycerides; TC, total cholesterol; WHR, waist to hip ratio*.

*P-values in bold indicate significance. The P-value was assessed using Student's t-test or Mann–Whitney U-test*.

### Subgroup Analysis in MAFLD Patients With Different Glucose Tolerance

**Figure 1:**
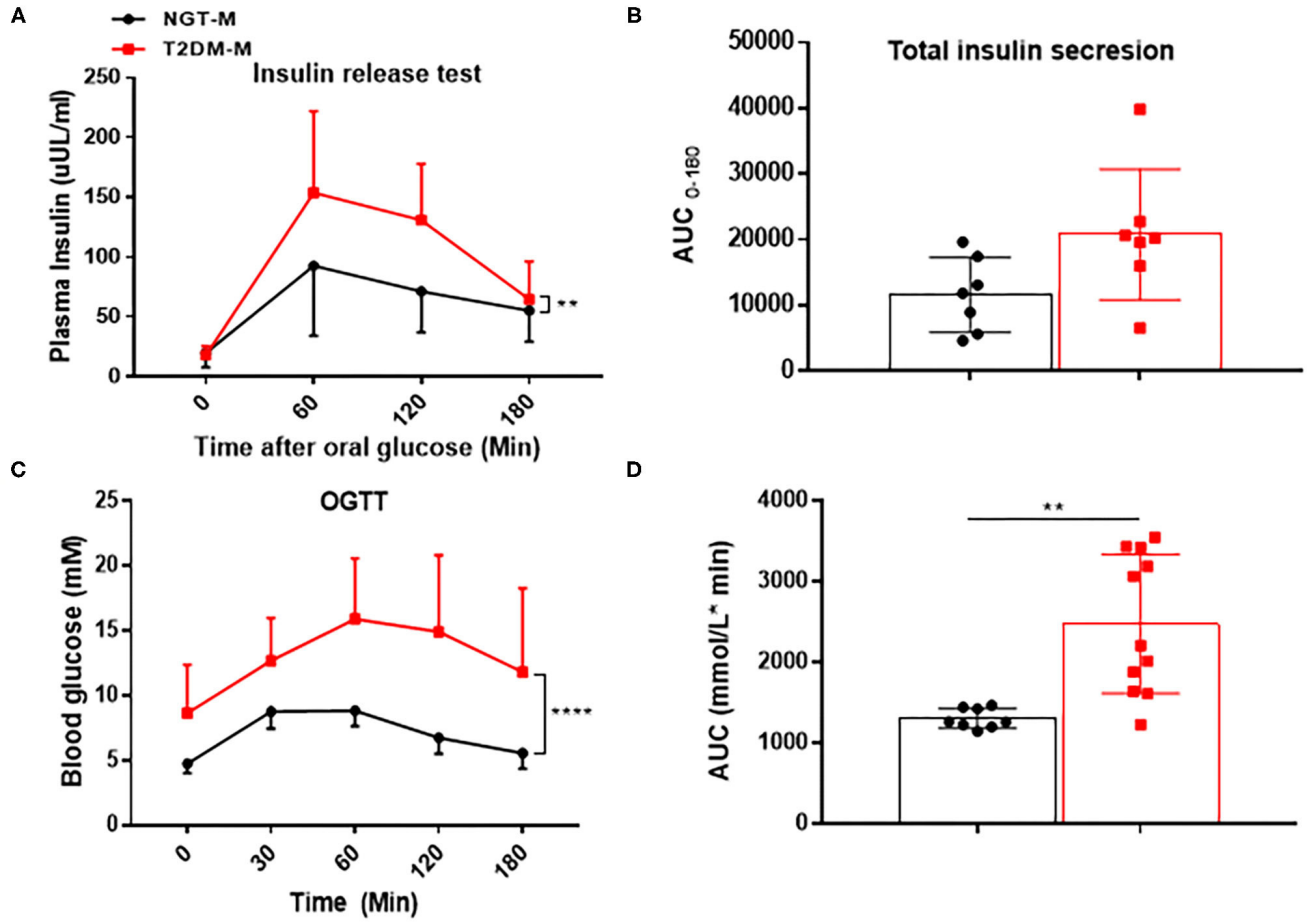
The indicators of insulin resistance in MAFLD patients. **(A,B)** Insulin release test and the respective analysis of the area under the curve (AUC) (*n* = 7). **(C,D)** Oral glucose tolerance test (OGTT) and the respective analysis of the area under the curve (AUC) (*n* = 8–11). The data are expressed as the mean ± SD, **P < 0.01, ****P < 0.0001.

To investigate the effect of abnormal glucose metabolism on adropin in MAFLD patients, we performed a subgroup analysis. As Table 2 showed, the two subgroups were similar in terms of age, BMI and WHR (All *p* > 0.05). However, the T2DM-M group had significantly higher levels of HbA_1_c, FBG, HOMA-IR, TG, and ALT than those of NGT-M group (All *p* < 0.05). Insulin release test and AUC suggested that insulin release peak at 1 h but not return to fasting level at 3 h in T2DM-M group (Figures 1A,B). OGTT and AUC showed that glucose tolerance was severely impaired in T2DM-M group compared with NGT-M subjects (Figures 1C,D). Notably, serum adropin level was significantly reduced in T2DM-M group compared with NGT-M group (0.51 ± 0.73 vs. 4.00 ± 3.52 ng/ml, *p* < 0.001).

**Table 2:** Anthropometric parameters and biochemical indexes among NGT and T2DM groups in MAFLD patients.

	**MAFLD**		***P-*value**
	**NGT (*n* = 13)**	**T2DM (*n* = 17)**	**NGT vs. T2DM**
Sex (male), *n* (%)	5 (38)	5 (29)	0.602
Age (years)	32.2 ± 9.5	37.3 ± 9.1	0.150
Weight (Kg)	110.90 ± 21.97	99.89 ± 17.09	0.132
BMI (Kg/m^2^)	38.66 ± 6.76	35.51 ± 4.67	0.133
Waist circumference (cm)	121.20 ± 12.9	110.9 ± 10.85	**0.025**
WHR	0.97 ± 0.06	0.94 ± 0.08	0.266
HbA_1_c (%)	5.36 ± 0.26	7.48 ± 2.18	**0.015**
FBG (mM)	5.54 ± 1.87	8.49 ± 2.86	**0.003**
FINS (μU/mL)	15.89 (12.09–19.47)	15.69 (13.23–23.90)	0.439
HOMA-IR	3.69 (2.20–4.38)	5.21 (4.18–9.64)	**0.020**
TC (mM)	4.62 ± 0.69	4.70 ± 0.79	0.820
TG (mM)	0.96 (0.78–1.86)	2.11 (1.83–3.69)	**0.037**
LDL-C (mM)	2.73 ± 0.57	2.81 ± 0.56	0.769
HDL-C (mM)	0.98 ± 0.21	0.85 ± 0.10	0.116
Apo-A1 (g/L)	1.46 ± 0.32	1.30 ± 0.09	0.138
Apo-B (g/L)	0.77 ± 0.19	0.85 ± 0.14	0.335
sd-LDL (mM)	0.68 ± 0.44	0.86 ± 0.46	0.415
NEFA (mM)	0.50 (0.40–0.50)	0.50 (0.48–0.63)	0.632
ALT (IU/L)	37.50 ± 14.85	65.40 ± 33.98	**0.047**
AST (IU/L)	47.25 ± 25.58	52.33 ± 26.88	0.640
GGT (IU/L)	41.43 ± 19.82	50.88 ± 49.86	0.647
Adropin (ng/ml)	4.00 ± 3.52	0.51 ± 0.73	**<0.001**
NAS	6.68 ± 1.61	6.94 ± 1.77	0.681

*Values are presented as mean ± SD (standard deviation of mean) for normally distributed data or medians (25th, 75th percentiles) for non-normally distributed data*.

*Apo-A1, apoprotein A1; Apo-B, apoprotein B; BMI, body mass index; FBG, fasting blood glucose; FINS, fasting insulin; HDL-C, high-density lipoprotein cholesterol; HOMA-IR, homeostasis model assessment of insulin resistance; LDL-C, low-density lipoprotein cholesterol; NAS, NAFLD activity score; NEFA, non-esterified fatty acid; sd-LDL, small dense-low-density lipoprotein cholesterol; TG, triglycerides; TC, total cholesterol; WHR, waist to hip ratio*.

*P-values in bold indicate significance. The P-value was assessed using Student's t-test or Mann–Whitney U-test*.

### Correlations of Serum Adropin Levels and Liver *ENHO* Expressions With Metabolic Parameters in MAFLD Subjects

**Figure 2:**
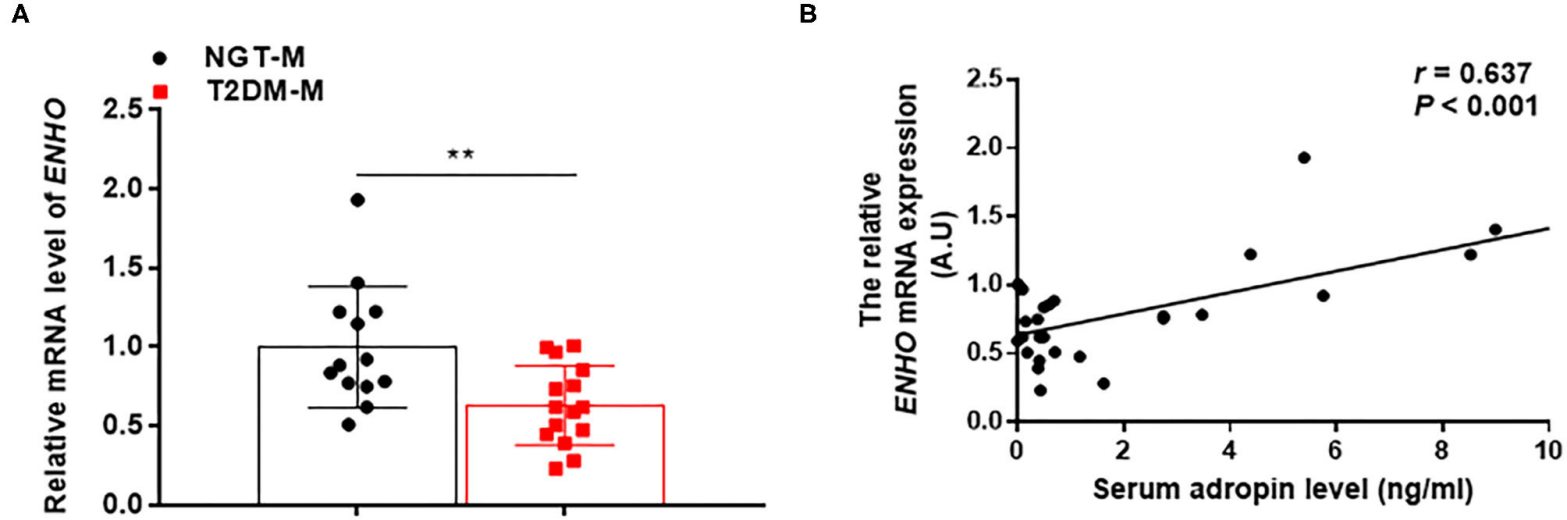
Adropin expression in the liver was lower in MAFLD subjects. **(A)** Quantitative real-time RT-PCR analysis of *ENHO* mRNA expression in the liver from MAFLD patients (*n* = 13–15). **(B)** Correlations of liver *ENHO* mRNA expression with serum adropin levels. The data are expressed as the mean ± SD, **P < 0.01.

To investigated the relationship between adropin and metabolic parameters in MAFLD patients, a linear correlation analysis was performed. As summarized in Table 3, serum adropin levels were negatively correlated with BMI, FBG, FINS, HbA1c and HOMA-IR, TG, Apo-B, NEFA, ALT, AST, and GTT, in spite of these differences did not reach the statistical significance. Interestingly, serum adropin levels were positively correlated with HDL-C and Apo-A1 (All *p* < 0.001). Furthermore, liver *ENHO* mRNA expression was obviously downregulated in T2DM-M group compared with NGT-M group (0.63 ± 0.06 vs. 1.00 ± 0.11, *p* < 0.01) (Figure 2A). Liver *ENHO* mRNA expression was positively correlated with Apo-A1 (*r* = 0.497, *p* < 0.05). Circulating adropin level was positively associated with liver *ENHO* mRNA expression (*r* = 0.637, *p* < 0.001; Figure 2B).

**Table 3:** Correlation of serum adropin level and liver *ENHO* mRNA expression with metabolic indicators in MAFLD patients.

**Variables**	**Serum adropin level (ng/ml)**		**Liver** ***ENHO*** **mRNA expression (A.U)**	
	***r***	***P***	***r***	***P***
BMI (Kg/m^2^)	−0.014	0.940	−0.016	0.937
Waist circumference (cm)	0.044	0.817	0.106	0.590
WHR	0.057	0.765	0.149	0.746
HbA_1_c (%)	−0.318	0.198	−0.218	0.385
FBG (mM)	−0.358	0.052	−0.152	0.439
FINS (μU/mL)	−0.166	0.510	−0.247	0.323
HOMA-IR	−0.308	0.215	−0.310	0.211
TC (mM)	0.190	0.450	−0.237	0.344
TG (mM)	−0.244	0.362	−0.352	0.182
LDL-C (mM)	0.018	0.944	−0.348	0.157
HDL-C (mM)	0.785	**0.000**	0.380	0.119
Apo-A1 (g/L)	0.780	**0.000**	0.497	**0.036**
Apo-B (g/L)	−0.007	0.976	−0.283	0.256
sd-LDL (mM)	0.128	0.625	−0.189	0.484
NEFA (mM)	−0.256	0.322	0.058	0.838
ALT (IU/L)	−0.369	0.131	−0.336	0.187
AST (IU/L)	−0.067	0.754	−0.008	0.971
GGT (IU/L)	−0.028	0.918	0.280	0.311

*Correlations between serum adropin or liver ENHO mRNA expression and anthropomorphic, blood chemistry data were calculated by Pearson's or Spearman's coefficient (r)*.

*Apo-A1, apoprotein A1; Apo-B, apoprotein B; BMI, body mass index; FBG, fasting blood glucose; FINS, fasting insulin; HDL-C, high-density lipoprotein cholesterol; HOMA-IR, homeostasis model assessment of insulin resistance; LDL-C, low-density lipoprotein cholesterol; NEFA, non-esterified fatty acid; sd-LDL, small dense-low-density lipoprotein cholesterol; TG, triglycerides; TC, total cholesterol; WHR, waist to hip ratio*.

*P-values in bold indicate significance*.

### Histological Features of the Liver in Patients With MAFLD

**Figure 3:**
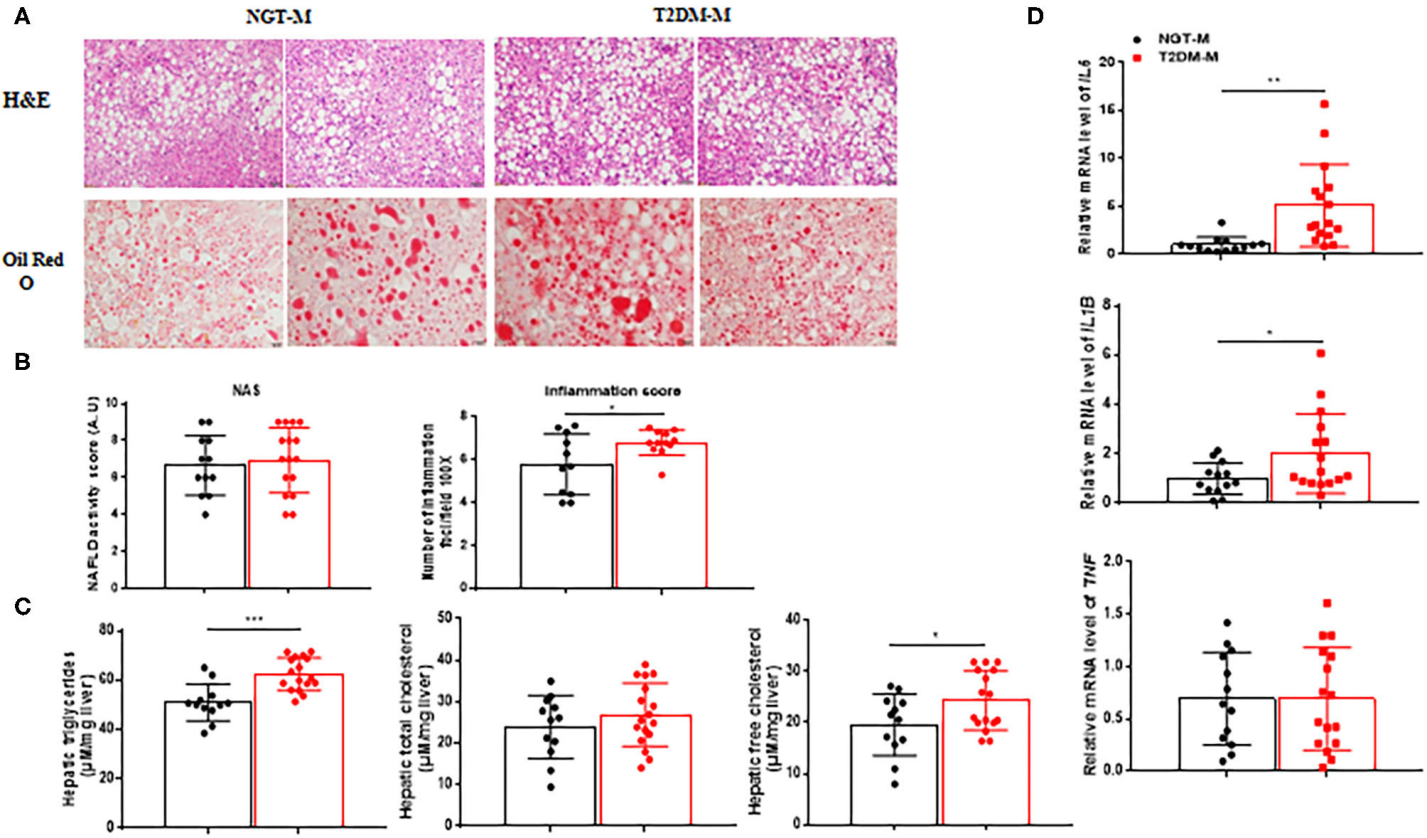
Steatohepatitis was exacerbated in T2DM-M group compared with NGT-M group. **(A)** Representative liver HandE staining (magnification, ×200; scale bar: 50 μm) and Oil Red O staining (magnification, ×400; scale bar: 20 μm) (*n* = 13–17). **(B)** Hepatic histological analysis of HandE staining (NAS and lobular inflammation score) (*n* = 11–16). **(C)** Hepatic triglycerides and (free) cholesterol levels (*n* = 12–17). **(D)** Quantitative real-time RT-PCR analysis of *TNF IL1B IL6* mRNA expressions in the liver from MAFLD patients (*n* = 13–16). The data are expressed as the mean ± SD, *P < 0.05, **P < 0.01, and ***P < 0.001.

**Figure 4:**
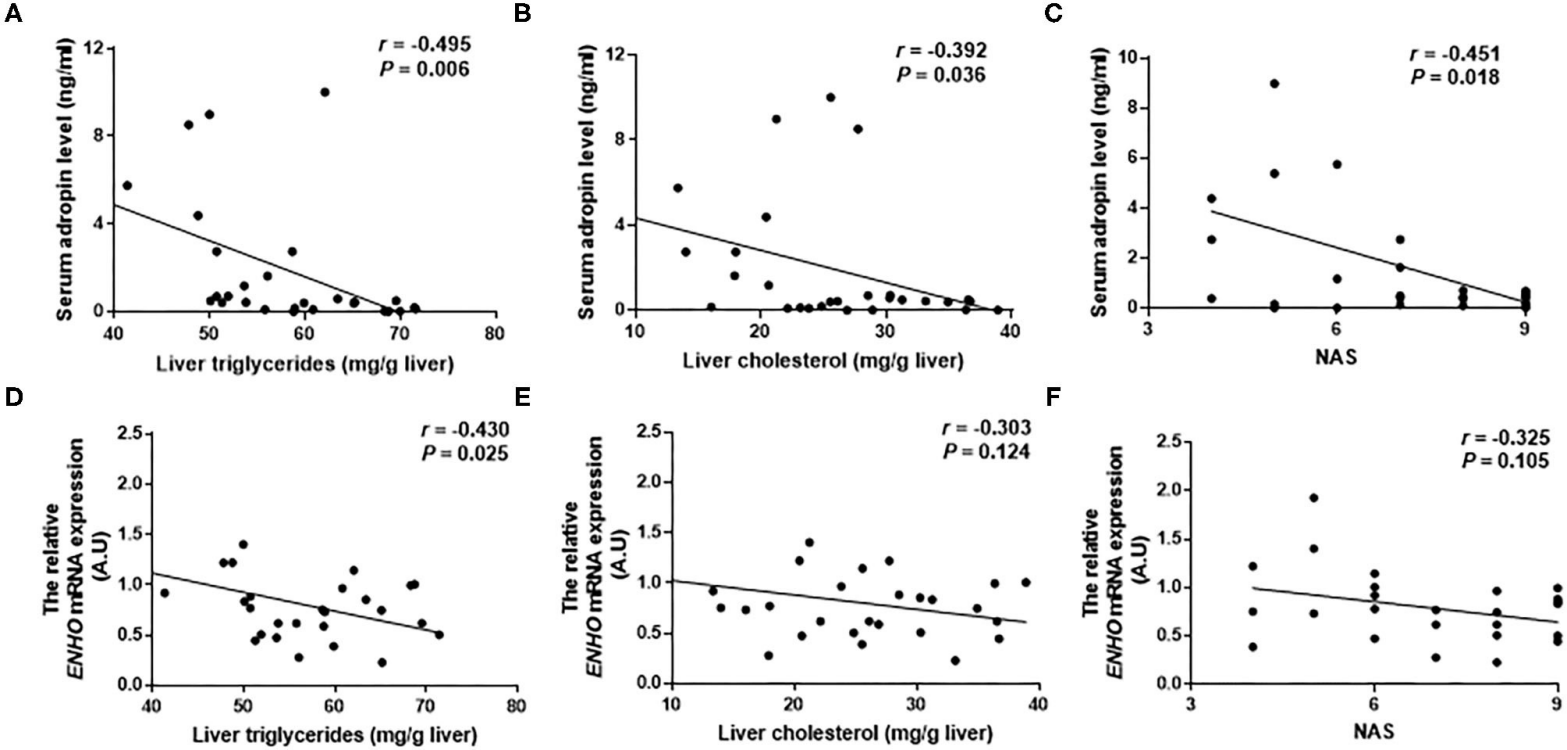
The correlation between serum adropin/liver adropin levels and the level of liver triglycerides **(A,D)**, and liver cholesterol **(B,E)**, and NAFLD activity score **(C,F)** in MAFLD patients.

T2DM-M patients exhibited more severe hepatic macrosteatosis, inflammation, and ballooning (Figure 3A), concurrent with a higher NAS and inflammation score (Figure 3B). Oil Red O staining demonstrated a more lipid accumulation in the T2DM-M patients (Figure 3A). Hepatic TG and free cholesterol were significantly higher in the T2DM-M group than those in the NGT-M group (Figure 3C). Moreover, the pro-inflammation genes (*TNF, IL1B*, and *IL6*) mRNA expression in the liver were higher in the T2DM-M group compared with NGT-M group (Figure 3D). Besides, serum adropin levels were significantly inverse correlated with intrahepatic TG (*r* = −0.495, *p* = 0.006; Figure 4A), TC (*r* = −0.392, *p* = 0.036; Figure 4B), and NAS (*r* = −0.451, *p* = 0.018; Figure 4C). Liver adropin levels were negatively correlated with intrahepatic TG (*r* = −0.430, *p* = 0.025; Figure 4D), TC (*r* = −0.303, *p* = 0.124; Figure 4E), and NAS (*r* = −0.325, *p* = 0.105; Figure 4F). We have analyzed the association between adropin and liver pro-inflammation genes. As shown in Supplementary Figures 1A–F, the mRNA expressions of *IL6* and *IL1B* were negatively correlated with adropin, but there were no statistically difference.

## Discussion

Our results indicate that serum adropin levels and liver *ENHO* mRNA expressions were significantly decreased in T2DM-M patients. These data verified the inverse association between circulating adropin and intrahepatic TG, TC, and NAS. As far as we known, few researches have been done to investigate the association of adropin with intrahepatic lipid contents in adult MAFLD patients. Overall, lower serum level of adropin may be a practical biomarker that can be used to detect the progression of MAFLD.

Several previous studies consistently showed that adropin played a critical role in maintaining glucose homeostasis, increasing the glucose utilization, improving glucose tolerance, and reducing insulin resistance (Butler et al., 2019; Jasaszwili et al., 2020). Adropin deficiency exacerbated the dysregulated glucose homeostasis and insulin resistance in diet-induced obesity (DIO) mice. Adropin injection significantly reduced fasting blood glucose, inhibited hepatic glucose production, lowered fasting insulin level, and improved insulin resistance in high-fat diet mice. *In vitro* study showed that adropin effects on glucose production were restricted in insulin resistance hepatocytes induced by palmitic acid or high glucose (Chen et al., 2017, 2020). Chronic consumption of high fat/high sucrose diets resulting in hepatic insulin resistance suppressed liver adropin expression, and could contribute to the dysregulation of glucose metabolism (Banerjee et al., 2020). Most clinical studies confirmed a dramatically decline of adropin in patients with T2DM when compared with healthy subjects (Wu et al., 2014; Chen et al., 2017). In accordance with prior studies, we found that serum adropin level and liver adropin expression was significantly decreased in T2DM-M patients. Moreover, serum adropin levels were positively correlated with liver adropin expression. Therefore, the lower serum adropin level of MAFLD patients may indicate more serious insulin resistance and glucose intolerance. All these data imply a closely connection between adropin and glucose homeostasis in MAFLD patients.

Adropin is required for metabolic homeostasis and is involved in preventing dyslipidaemia (Kumar et al., 2008). Animal studies suggested that adropin could suppress fatty acid oxidation (Gao et al., 2014). In human subjects, circulating adropin was negatively correlated with the levels of plasma TG, apolipoprotein B, and LDL-c and was positively correlated with the HDL-c level (Butler et al., 2012). Our findings are consistent with previous studies that serum adropin concentrations were inversely associated with these indices of blood lipid. It is important to note that liver TG and TC was inversely correlated with serum adropin level, but only liver TG was significantly negative correlated with liver *ENHO* mRNA expression. We believed that two reasons might explain these differences. On the one hand, circulating adropin levels may associate with the discrepancy of the equilibrium between cholesterol synthesis and processing of circulating lipoprotein particles (Ghoshal et al., 2018). On the other hand, liver *ENHO* mRNA expression is regulated by the biological clock and nutrition status. Experiments based on mice overexpressing adropin unable to prove the role for adropin in regulating cholesterol uptake from the diet, clearance from the circulation or cholesterol biosynthesis (Ghoshal et al., 2018). The precise effect of adropin in lipid metabolism is still unclear. Further studies examining the relationship between adropin expression in liver tissues and intrahepatic TG and TC are required. Our study showed that circulating adropin was negatively correlated with intrahepatic TG and TC.

Genetically engineered animals provided strong evidence indicating that adropin contributes to the modulation of inflammation. Chen et al. (2019) demonstrated that serum adropin concentrations were obviously decreased in steatohepatitis, adropin deficient mice showed exacerbated hepatic steatosis, inflammation and fibrosis when fed methionine-choline deficient (MCD) or western diets. Serum adropin level was lower in B-ultrasound or liver biopsy diagnosed NAFLD patients, and was associated with the severity of NAFLD (Sayin et al., 2014; Kutlu et al., 2019). Adropin treatment decreased the expression of the proinflammatory cytokines *Il1b, Il6*, and *Tnfa* in mice with MCD diet-induced steatohepatitis (Chen et al., 2019). Thus, adropin as a potential anti-inflammatory factor plays an important role in the process of steatohepatitis. Herein, we included patients with biopsy-proven hepatic steatosis to explore the association between adropin and liver injury. We found that both serum adropin level and liver *ENHO* mRNA expression were negatively associated with ALT and NAS. The proinflammatory genes (*TNF, IL6*, and *IL1B*) were increased in T2DM-M patients compared with those in NGT-M group. In addition, the serum adropin levels and liver *ENHO* mRNA expressions were dramatically decreased in T2DM-M patients compared with NGT-M group. Thus, our findings pointed out the potential of adropin in identifying MAFLD, especially in T2DM individuals.

A limitation of this study is the cross-sectional design which harbored a small sample size, and the liver samples from patients who volunteered to have a liver biopsy, which may lead to selection bias. Hence, more large and random liver-biopsy hepatic steatosis populations are needed to further confirm our results and conclusions.

## Conclusions

To sum up, the current findings provide further indication of the significance of adropin in maintaining metabolic homeostasis. Adropin may participate in the pathogenesis of MAFLD and can be served as a predictive biomarker of MAFLD. Nevertheless, more *in vivo* experiments and a large-scale clinical trial is required to confirm these data in MAFLD.

## Data Availability Statement

The original contributions presented in the study are included in the article/Supplementary Material, further inquiries can be directed to the corresponding author/s.

## Ethics Statement

The studies involving human participants were reviewed and approved by the Ethics Committee of the China-Japan Friendship Hospital (2019-103-K71). The patients/participants provided their written informed consent to participate in this study.

## Author Contributions

GW, JL, HM, and AQ: conceived and designed the experiments. NL, GX, and AQ: analyzed the data. NL, GX, AQ, and BZ: contributed reagents materials analysis tools. NL: wrote the paper. GW, JL, and AQ: revised and adapted the manuscript. All authors read and approved the final manuscript.

## Conflict of Interest

The authors declare that the research was conducted in the absence of any commercial or financial relationships that could be construed as a potential conflict of interest.

## Abbreviations

Apo-A1: apoprotein A1
Apo-B: apoprotein B
BMI: body mass index
ELISA: enzyme-linked immunosorbent assay
ENHO: energy homeostasis associated gene
FBG: fasting blood glucose
FINS: fasting insulin
FFAs: free fatty acids
HbA1c: glycated hemoglobin
HDL-C: high-density lipoprotein cholesterol
HOMA-IR: homeostasis model assessment of insulin resistance
LDL-C: low-density lipoprotein cholesterol
MAFLD: metabolic dysfunction-associated fatty liver disease
MCD: methionine-choline deficient
NAFLD: nonalcoholic fatty liver disease
NAS: NAFLD activity score
NEFA: non-esterified fatty acid
NGT: normal glucose tolerance
OGTT: oral glucose tolerance test
sd-LDL: small dense-low-density lipoprotein cholesterol
T2DM: type 2 diabetes mellitus
TG: triglycerides
TC: total cholesterol
WHR: waist to hip ratio.
