# Supplementary figures and images for: Serum Adropin as a Potential Biomarker for Predicting the Development of Type 2 Diabetes Mellitus in Individuals With Metabolic Dysfunction-Associated Fatty Liver Disease
Source: Front Physiol. 2021 Jul 22;12:696163. doi: 10.3389/fphys.2021.696163 (PMC8339918; doi:10.3389/fphys.2021.696163)

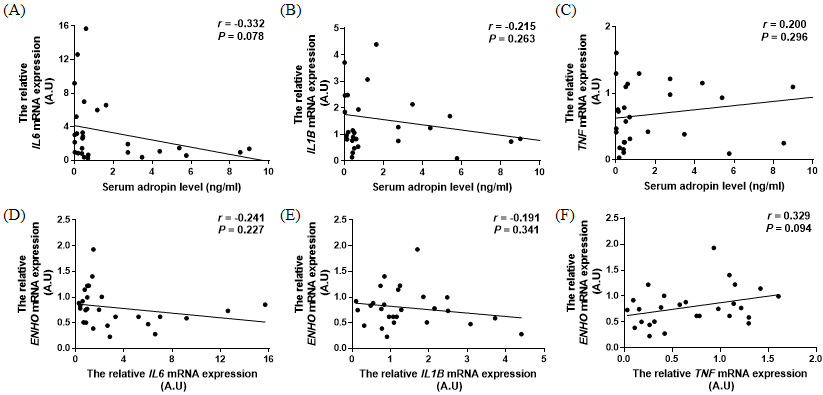

Supplement: Supplementary file 1 [file Image_1.TIF]
