# Supplementary material for: Serum Adropin as a Potential Biomarker for Predicting the Development of Type 2 Diabetes Mellitus in Individuals With Metabolic Dysfunction-Associated Fatty Liver Disease
Source: Front Physiol. 2021 Jul 22;12:696163. doi: 10.3389/fphys.2021.696163 (PMC8339918; doi:10.3389/fphys.2021.696163)
Supplement: Supplementary file 2 [file Table_1.DOCX]

**Supplemental Table 1. List of primer sequences for qPCR analysis**

| **Genes** | **NCBI accession No.*** | **Forward** | **Reverse** |
| --- | --- | --- | --- |
| *ACTB* | NM_001101.5 | 5’-GAGCACAGAGCCTCGCCTTT-3’ | 5’-TCATCATCCATGGTGAGCTGG-3’ |
| *ENHO* | NM_198573.3 | 5’-AGGCTCAACTCAGGCTCAGG-3’ | 5’-CTGTCTGCACGCTCAGTGAT-3’ |
| *TNF*  *IL1B*  *IL6* | NM_000594.4  NM_000576.3  NM_000600.5 | 5’-GCACTTTGGAGTGATCGGC-3’  5’-AGCCATGGCAGAAGTACCTG-3’  5’-GGCACTGGCAGAAAACAACC-3’ | 5’-CCTCAGCTTGAGGGTTTGCTAC-3’  5’-CCTGGAAGGAGCACTTCATCT-3’  5’-CACCAGGCAAGTCTCCTCAT-3’ |

*https://www.ncbi.nlm.nih.gov/nuccore/; January 31, 2021.
